# A Molecularly Imprinted Membrane for High-Density Lipoprotein Extraction in Point-of-Care Testing

**DOI:** 10.3390/bios15100685

**Published:** 2025-10-10

**Authors:** Gian Luca de Gregorio, Denis Prim, Alberto Zavattoni, Italo Mottini, Daniele Pezzoli, Federico Roveda, Marc E. Pfeifer, Jean-Manuel Segura

**Affiliations:** 1 Institute of Life Sciences-School of Engineering, HES-SO/University of Applied Sciences and Arts Western Switzerland, 1950 Sion, Switzerland; gianluca21@hotmail.it (G.L.d.G.); denis.prim@hevs.ch (D.P.); marc.pfeifer@hevs.ch (M.E.P.); 2 PRIMA Lab SA, 6828 Balerna, Switzerland

**Keywords:** point-of-care testing, cholesterol, lipoprotein, enzymatic assay, molecularly imprinted polymer, molecularly imprinted membrane, solid-phase affinity extraction, nanoparticle

## Abstract

Cholesterol blood levels in low-density lipoproteins (LDLs) are a key parameter for assessing the risk of cardiovascular diseases. Direct quantification of LDL cholesterol at the point of care would be possible if all other lipoproteins, particularly the high-density lipoproteins (HDLs), could be removed prior to measurement. Here, we investigated whether a molecularly imprinted membrane (MIM) could be used for the solid-phase affinity extraction (SPAE) of HDL in a paper-based lateral flow test. Samples traveled by capillarity through the MIM before reaching a detection zone where LDL cholesterol was quantified enzymatically. MIMs were produced by impregnation of the membrane with a dispersion of molecularly imprinted polymers (MIPs) selective for HDL. MIPs were synthesized using precipitation polymerization and exhibited good selectivity for HDL compared with LDL and an uptake capacity of 5.0–7.0 µg of HDL-C/mg of MIP. The MIM enabled the removal of HDL with an efficiency of typically 68%. However, quantification of LDL cholesterol suffered from strong non-specific binding of LDL, likely due to its inherent colloidal instability. Overall, our results highlight the challenges associated with SPAE of colloidal particles. Furthermore, our study demonstrates a novel, efficient, and potentially generic modality to integrate SPAE into paper-based POC diagnostic tests.

## 1. Introduction

Cardiovascular diseases are considered to be the most common non-communicable diseases globally, with an estimated 17.8 million deaths in 2017 [1]. Coronary artery disease consists of a build-up of atherosclerotic plaques in the vasculature. The formation of these plaques is closely related to the levels of total cholesterol and levels of cholesterol in low-density lipoproteins (LDLs) [2,3]. The determination of LDL cholesterol (LDL-C) levels in blood is therefore a key parameter to assess and prevent the risk of developing cardiovascular diseases [3]. To enable more frequent decentralized testing and timely intervention, cholesterol is increasingly being measured at the point of care (POC) using rapid diagnostic tests [3,4,5] such as paper-based tests [6,7,8,9]. However, the selective analysis of LDL-C is complex to realize as LDL coexists in blood with several other classes of lipoproteins that differ from each other by their size, density, and composition. The most important of those are LDL and high-density lipoproteins (HDLs), as they contain most of the cholesterol, with 60–70% in LDL (LDL-C cholesterol) and 20–30% in HDL (HDL-C cholesterol). Therefore, the direct determination of LDL-C requires either using a selective method of analysis or, as a novel approach investigated here, the prior removal of the other lipoproteins, in particular HDL, using solid-phase affinity extraction (SPAE).

Molecularly imprinted polymers (MIPs) are a class of highly cross-linked polymer-based molecular recognition elements that can be used as active elements in sensing and in SPAE [10,11,12]. MIPs are engineered to bind one target compound or a class of structurally related target compounds with high selectivity. Selectivity is introduced during MIP synthesis thanks to a template molecule that guides the formation of specific cavities or imprints that are sterically and chemically complementary to the target analyte, which can be a small molecule, a protein, or even a cell. Currently, the only available publications reporting MIPs for the selective binding of lipoproteins, in particular HDL and LDL, are the works of S. Chunta and P. A. Lieberzeit [13,14,15,16]. In these studies, MIP surfaces covering an electrode were produced by imprinting a template-patterned stamp on the surface of a polymerizing film having a suitable monomer composition. This strategy proved successful for the realization of an LDL-C sensor based on a quartz crystal microbalance detection [14]. As an alternative approach, MIPs selective for HDL could be used to develop an SPAE element integrated in a POC test, enabling the use of other detection means for LDL-C quantification, such as a colorimetric enzymatic readout.

MIPs have already been used in multiple POC paper-based analytical and diagnostic tests as sensing elements [17,18] and in a few articles as SPAE elements (e.g., [19]). A modality for integrating MIPs for SPAE in paper-based POC testing is molecularly imprinted membranes (MIMs) [20,21,22]. MIMs can be commonly produced by polymerization (in situ polymerization, phase-inverse polymerization, emulsion polymerization) of an appropriate monomer mix in the presence of the template with a thickness control mechanism, such as the squeezing between two plates to obtain a thin sheet of MIP. These membranes, however, may exhibit low porosity. Alternative common approaches are to prepare MIM by electropolymerization at the surface of an electrode or to polymerize MIPs inside or at the surface of porous framework structures. This last approach enables the incorporation of MIM into porous materials compatible with POC diagnostic assays, such as filter paper [23], regenerated cellulose [24], cellulose acetate [25], nanocellulose [26], glass fibers, and nitrocellulose [27]. Finally, MIM can also be produced by electrospinning a solution of a spinning fiber material, such as cellulose acetate, with MIP nanoparticles [28] or by chemically grafting MIP nanoparticles to a cellulose membrane [29]. However, these different approaches result in constraints on the nature of the monomers, surface chemistry, and/or polymerization process that may be incompatible with the imprinting of complex targets.

**Figure 1:**
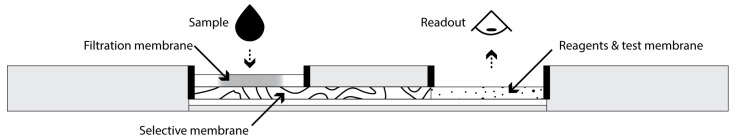
Side cross-sectional view of a potential implementation of a POC analytical device integrating SPAE for quantitative analysis of non-HDL cholesterol. Typical dimensions of the device could be 7 cm length × 2 cm width × 0.5 cm height.

In this study, we aimed to develop and characterize an SPAE MIM integrated within a POC paper-based analytical device to efficiently remove HDL from serum samples for subsequent selective analysis of non-HDL cholesterol, particularly LDL-C, using a colorimetric enzymatic assay. Figure 1 depicts a possible implementation of this idea for POC testing. To this aim, MIP nanoparticles able to selectively and efficiently bind HDL were synthesized and then incorporated in a paper membrane using a novel precipitation approach to yield MIMs. The test was operated in a lateral flow format with the serum sample flowing through the MIM before reaching a detection zone. The performance of the MIM for capturing HDL and its selectivity for HDL compared with LDL were evaluated, and the potential of this approach as an SPAE-based platform for POC diagnostic solutions was investigated.

## 2. Materials and Methods

### 2.1. Chemical Reagents

Methacrylic acid 99% (MAA), 1-vinyl-2-pyrrolidone 99% (VP), *N*,*N*-(1,2-dihydroxyethylene)bisacrylamide 97% (DHEBA), *N*,*N*,*N*’,*N*’-tetramethylethylenediamine 99% (TEMED), horseradish peroxidase (HRP), cholesterol oxidase (CO), Nile Red (for microscopy) and 2,2′-Azino-bis(3-ethylbenzothiazoline-6-sulfonic acid) diammonium salt 98% (ABTS), Tween 20, were purchased from Sigma-Aldrich (St-Louis, MO, USA); ammonium persulfate 98% (APS) and bovine serum albumin fraction V (BSA) was purchased either from Sigma-Aldrich or Carl Roth (Karlsruhe, Germany); high-density lipoprotein (HDL, 3040–3510 mg cholesterol/dL), delipidated serum, and low-density lipoprotein (LDL, 3570 mg cholesterol/dL) solutions from human plasma were purchased from MyBiosource (San Diego, CA, USA) or Lee BioSolutions (Espoo, Finland). Cholesterol esterase (CE) was purchased from AG Scientific (San Diego, CA, USA). Glacial acetic acid 99–100% was purchased from J.T. Baker (Thermo Fisher Scientific Inc., Waltham, MA, USA). Reagents were used as received with no further purification.

Phosphate buffer saline solution (PBS 10×) was purchased from Gibco (Thermo Fisher Scientific Inc., Waltham, MA, USA) and diluted to the desired concentration with MilliQ water. The resulting PBS solution was filtered through a 0.2 µm filter and stored at 4–8 °C for no more than one month.

### 2.2. Synthesis of MIP Nanoparticles and Nanoparticle Aggregates

MIP nanoparticles were synthesized as follows: A total of 46.9 mg of DHEBA (234 mmol) was weighed in a 40 mL cylindrical glass gas-tight reaction vessel and dissolved in 9.41 mL of PBS 5× (concentration of 4.98 g/mL) with an ultrasonic bath. After complete solubilization of DHEBA, 116 µL of a 10.0% *v*/*v* VP solution in PBS 5× (12.06 mg, 0.104 mg/mL) and 79.2 µL of a 10% *v*/*v* MAA solution in PBS 5× (8.04 mg, 0.101 mg/mL) were added. The total monomer content was 0.67% *w*/*v*, and the ratio between monomers was DHEBA/VP/MAA = 70/18/12, defined as weight percentage. The pH of the solution was adjusted to 6.0–6.5 using HCl 6 M or NaOH 5 M. The resulting solution was quickly vortexed at the maximum rate for a few seconds. The solution was purged under stirring (600 rpm) with inert gas (Ar or N_2_) for at least 15–20 min. Then, 335 µL of HDL template solution 1.887 g/dL in cholesterol (the mass of cholesterol corresponded to 10% weight of the total mass of monomers) was added. The stir rate was reduced to 230 rpm to avoid excessive foaming, and the solution was purged with inert gas for 15 min. The mixture was left under stirring for five to six hours at room temperature. The reaction vessel was then purged with inert gas for 10 min and 5.57 µL of TEMED, followed by 5.57 mg of APS dissolved in 30 µL of PBS 5× being quickly added. Mechanical stirring was immediately stopped. The solution was quickly homogenized with a vortex for a few seconds and purged for 10 min with inert gas, and the polymerization was allowed to proceed for 16–17 h at room temperature with no stirring. Once the reaction was completed, the solution of MIP nanoparticles appeared cloudy with no or minimal precipitate present.

To remove the HDL template and produce the MIP nanoparticle aggregates, 1.10 mL of glacial acetic acid was added, and the solution was vigorously mixed for five minutes. The solution was then transferred into a 15 mL centrifuge tube and centrifuged at 21,900× *g* (15,000 rpm) for 20 min. The MIP nanoparticle aggregates (MIP NPAs) precipitated as a white/transparent gel, and the supernatant was discarded. The precipitated MIP nanoparticle aggregates were then redispersed in 5.0 mL of milliQ water and reprecipitated by centrifugation at 1920× *g* (4500 rpm) for 10 min. The washing procedure with milliQ water was performed at least three additional times; then, the recovered MIP nanoparticle aggregates were freeze-dried overnight and collected as a white/yellowish powder, which was insoluble and poorly dispersible in most common solvents (water, ethanol, acetonitrile, acetone, toluene, etc.).

To produce non-imprinted nanoparticles and nanoparticle aggregates, the procedure was the same as for the MIP NPAs except that no HDL was added.

### 2.3. Lipoprotein Depletion Experiments in Solution

The HDL-C uptake capacity of MIP NPAs was evaluated by mixing 3.0 mg of MIP NPA with 500 µL of PBS 1× buffer (concentration of 6.0 mg/mL in MIP NPA) containing 50 µg of HDL-C (concentration of 10 mg/dL in HDL-C) in a 2 mL Eppendorf vial. HDL-C uptake capacity was only evaluated at a pH of 7.4 and at room temperature as these were the conditions planned for the practical use of test. The mixture was agitated using a Stuart rotator set at 40 rpm to ensure proper diffusion of the target to the MIP NPAs. After the desired time (typically 1 h), the vial was centrifuged at 5000 rpm for 60 s to ensure complete precipitation of the MIP NPAs. The resulting supernatant was analyzed using an enzymatic assay to determine the amount of unbound HDL-C. The difference between the starting HDL-C concentration and the final HDL-C concentration yielded the amount of bound HDL-C. The amount of bound HDL-C increased linearly with the amount of MIP NPA used.

The enzymatic reagent solution for the determination of cholesterol in solution was prepared by mixing 100 µL of CE at 20 U/mL in PBS 1×, 20 µL of CO at 77 U/mL in PBS 1×, 20 µL of HRP at 100 U/mL in PBS 1×, and 100 µL of 15 mM ABTS in citric buffer pH 5.2, with 760 µL of citric buffer pH 5.2. The amount of unbound cholesterol in the solution was measured by dissolving 12.5 µL of the sample solution with 37.5 µL of PBS 1× and 50 µL of the enzymatic reagent solution. The resulting solution was incubated at 37 °C for 30 min, and the absorbance was measured at 405nm with a SpectraMAX microtiter plate reader (Molecular Devices, CA, USA). Samples containing 6.25 µL, 12.5 µL, and 25 µL of a 10 mg/dL HDL-C solution were diluted, respectively, with 43.75 µL, 37.5 µL, and 25 µL of PBS 1× and 50 µL of the enzymatic reagent solution and used as reference standards. Each sample was measured in duplicate, and the average value was calculated. Blank solutions were prepared by mixing 50 µL of PBS 1× with 50 µL of the enzymatic reagent solution.

The specificity of MIP NPAs for HDL compared with LDL was evaluated with the same method, except that 50 µg of LDL-C was used instead of HDL-C. The addition of bovine serum albumin (BSA, 1%) to the solution enabled minimization of the adsorption of LDL to the walls of the vial.

### 2.4. Characterization of MIP Nanoparticles and Nanoparticle Aggregates

Dynamic light scattering experiments were performed on a Zetasizer Nano ZS instrument (Malvern Instruments Ltd., Malvern, UK), working at 633 nm and equipped with a backscatter detector (173°). Samples were analyzed in a PMMA disposable cuvette (Brand). Measurements were performed using a 173° backscatter angle of detection. Samples were previously equilibrated to 20 °C for ten minutes and were analyzed in triplicate. Each measurement consisted of 11 runs of 10 s. Data were analyzed using a general-purpose model with normal resolution, using for the polyplex a refractive index of 1.35 and an absorption of 0.01; and for the formulation buffer a viscosity of 1.002 mPa·s and a refractive index of 1.330.

### 2.5. Preparation and Characterization of Molecularly Imprinted Membranes

Molecularly imprinted membranes (MIMs) for HDL were prepared by depositing 42 µL (7 depositions, 6 µL each) of a suspension of MIP nanoparticles, prepared as in Section 2.2, to an 8 cm strip of chromatographic paper (Whatman chromatographic paper CHR01, Cytiva, Marlborough, MA, USA) or dipstick paper (Cytiva CF1 paper, Cytiva, USA). The chromatographic paper was allowed to dry out after each deposition on a heating plate set at 50 °C. Only a specific area of the strip was impregnated (MIM zone). The membrane was then immersed in a 10% *v*/*v* acetic acid bath for 5 min, followed by four washes in deionized water baths until the resulting pH was neutral. The strip was left on a heating plate set at 50 °C until complete drying, with weights placed over it to prevent folding. Imaging of the MIP NPAs attached to cellulose fibers was carried out by scanning electron microscopy on a FEI Teneo instrument, equipped with an in-lens (Trinity) detector operated at 2.00 kV (25 pA). The sample was coated with a 5 nm layer of iridium before imaging.

For the analysis of lipoprotein SPAE, 1.3 µL of an HDL or LDL solution in PBS at a concentration of 5.77 mg/mL (in total 7.5 µg HDL-C or LDL-C) was added as a spot on a line in front of the MIM zone and allowed to dry at ambient conditions. For fluorescence imaging using Nile Red, 49.5 µL of the HDL or LDL solution at 5.77 mg/mL was spiked with 0.5 µL of a 10 mM Nile Red solution in dimethyl sulfoxide (DMSO) prior to deposition. The front end of the sample deposition zone of the strip was then immersed in a well containing PBS buffer. The capillary flow of buffer was stopped when it almost reached the end of the strip.

For Nile Red imaging, the paper strip was left to dry under ambient conditions, and then the fluorescence intensity was examined with a luminescence imaging system (Vilber FX, filter set ex 650nm, em 695nm, Marne-la-Vallée, France) with an exposure time of 240 ms. The obtained images were processed and analyzed using ImageJ (version 1.53k), a public domain software developed by the U.S. National Institutes of Health (Bethesda, MD, USA), to obtain a fluorescence profile along the paper strip by integrating the fluorescence intensity over the entire width of the paper strip. The background signal was evaluated by recording fluorescence images of the paper strip before a run under the same illumination conditions. The background signal was subtracted from the measurement signal for the quantitative analysis of the obtained data.

For imaging using the enzymatic assay, after elution, the integrated strips were sprayed with a 3× concentrated reagent enzymatic solution using a spray dispenser (BioDot, Irvine, CA, USA) and left to dry out under ambient conditions inside a fume hood. The images of the strips after reaction with the enzymatic reactive solution were acquired with a Vilber FX imaging system (exposure time of 240 ms).

### 2.6. Statistical Analysis

Data were analyzed using Excel (Microsoft, Seattle, WA, USA), Origin (OriginLab, Northampton, MA, USA), and Igor (Wavemetrics, OR, USA). Error bars are ±1 standard deviation.

## 3. Results and Discussion

### 3.1. Synthesis of MIP Nanoparticles and Nanoparticle Aggregates

To produce MIP nanoparticles against HDL in aqueous buffers, the monomers methacrylic acid (MAA), 1-vinyl-2-pyrrolidinone (VP), and *N*,*N*′-(1,2-dihydroxyethylene)-bisacrylamide (DHEBA, cross-linker) were used in a weight ratio DHEBA/VP/MAA = 70/18/12, as in the work of Chunta and Lieberzeit [8]. To verify whether these monomers were appropriate for producing MIPs against HDL, an affinity screening was performed (see ESI section A and Table S1), which confirmed that MAA and VP display a strong affinity for HDL, with only 4-vinylpyridine (4-VP) exhibiting a similar interaction. The performance of MIPs produced with 4-VP as a monomer was, however, not improved compared with the use of VP.

**Figure 2:**
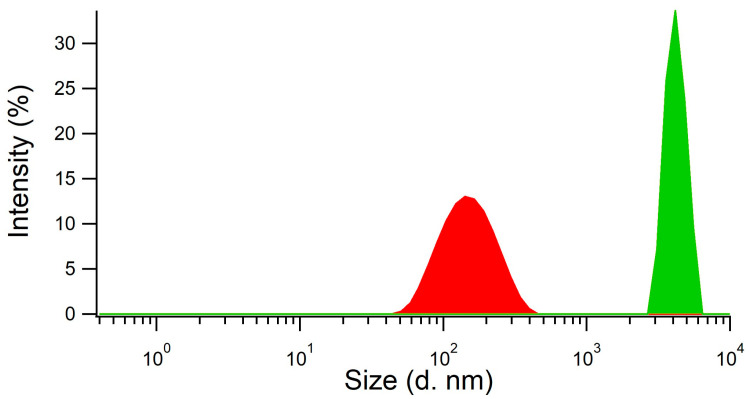
MIP particle aggregates were formed by the coalescence of metastable MIP nanoparticles upon addition of acetic acid, as shown by measurements of particle size distribution using dynamic light scattering. MIP nanoparticles (red distribution) had an average size of 140–160 nm and the aggregates a size of several µm (green distribution).

We did not assess the synthesis approach used by Chunta and Lieberzeit [8] as directly applicable to the depletion of HDL within membranes because it was designed to produce imprinted surfaces for sensing. Therefore, a new synthetic procedure consisting of a ‘precipitation polymerization’ was developed to produce HDL imprinted polymer nanoparticles. The reaction was performed in two steps, with the first step being the preparation of the mixture of monomers and template HDL, and the second step the polymerization conducted in the absence of stirring after the addition of the catalyst (TEMED) and the initiator (APS). The obtained product was a metastable cloudy dispersion of imprinted nanoparticles with an average size of 140–160 nm, as measured by dynamic light scattering (DLS, Figure 2, red curve). The addition of acetic acid for template removal resulted in the concomitant precipitation of the nanoparticles as nanoparticle aggregates (NPAs) with a size of several µm (Figure 2, green curve). The MIP NPAs obtained were recovered in the form of a poorly dispersible powder.

The use of a small total monomer content below 0.80% *w*/*v* was instrumental in preventing gelation of the solution. pH and salt concentration played a key role in determining the yield of MIP synthesis. Yields of typically 90% were obtained when using a pH ranging between 6.0 and 6.5 and a sodium chloride concentration of at least 0.68 M. Much poorer yields were obtained when the reaction was performed at low salt concentration or at other pH values. These conditions were similar to those used in the article by Chunta and Lieberzeit [8], where HDL was in a solution containing at least 1 M of NaBr. The high amount of salt should result in the strong attenuation of electrostatic interactions, indicating that the interaction between HDL and the MIP was rather mediated by hydrophobic interactions and possibly H-bonding.

### 3.2. Characterization of the Properties of the MIP NPA

**Figure 3:**
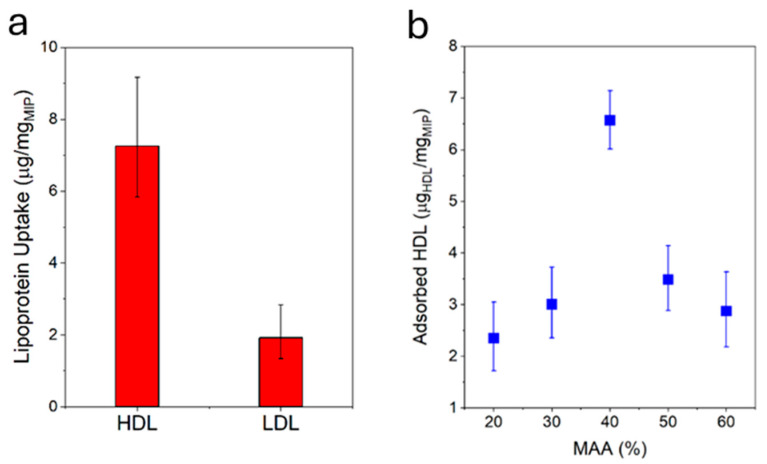
(**a**) MIP particle aggregates were specific for HDL, as demonstrated by the higher uptake of HDL compared with LDL under identical conditions. Target uptake was assessed by incubating MIP NPAs with either HDL or LDL for 1h and measuring the difference in lipoprotein concentration in the solution prior to and after incubation using an enzymatic assay. (**b**) HDL-C binding capacity after 1h as a function of the ratio between MAA/VP in MIP NPA. The optimal ratio was between 40 and 50% MAA.

The HDL-C uptake capacity of the MIP NPA was evaluated by mixing defined quantities of MIP NPAs and HDL-C and, after varying periods of time, centrifuging down the MIP NPAs to quantify the depletion of HDL-C in the supernatant using an enzymatic assay. MIP NPAs were capable of adsorbing an amount of ~6.5–7.0 µg of HDL-C/mg of MIP after one hour of incubation (Figure 3a) and up to ~11.7 µg of HDL-C/mg of MIP after five hours. As typical concentrations of HDL-C in serum are ~50 mg/dL [2,3], a 25 µL blood drop should contain ~7.5 µg of HDL-C. The binding sites in ~1.0 mg MIP NPAs should therefore be adequate for the envisaged application.

The specificity of MIP NPAs for HDL compared with LDL was evaluated by performing an uptake assay of LDL-C. In the worst case, LDL-C uptake was observed to be ~2.0–3.0 µg of LDL-C/mg of MIP (Figure 3a) and was partly due to adsorption to the vial walls, as shown by measurements in the absence of MIP NPAs. Given that typical values of LDL-C in serum are ~130 mg/dL, i.e., ~20 µg of LDL-C in 15 µL of serum, 1.0 mg of MIP NPA would be expected to roughly deplete only ~2.0 µg of LDL-C, i.e., the recovery of LDL-C should be around 90%.

The chemical composition of the MIP NPAs in terms of ratios between the two monomers MAA and VP was an essential parameter in determining HDL binding capacity and specificity. The optimal ratio was between 40% and 50% of MAA with a relatively steep dependency (Figure 3b), and it corresponded to the observations reported in previous publications [8].

### 3.3. Integration of MIP NPAs in Paper Membranes and Characterization

**Figure 4:**
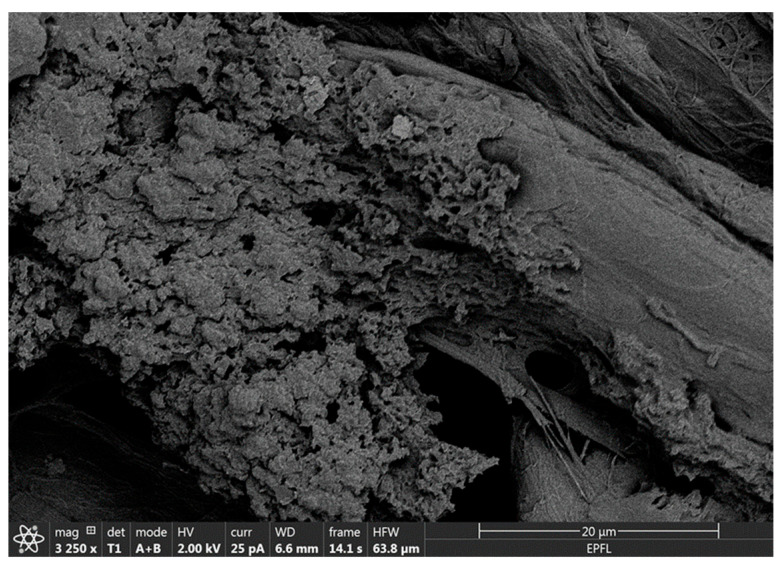
Scanning electron microscopy image of MIP NPAs attached to cellulose fibers in an MIM specific for HDL. MIMs were produced by impregnating a chromatographic paper membrane with a suspension of MIP nanoparticles, followed by aggregation and precipitation within the membrane using acetic acid.

MIP NPAs were a poorly dispersible powder that, in this form, could not be integrated into membranes to produce molecularly imprinted membranes (MIMs). However, at an intermediate step of the synthetic process, after the polymerization and before template removal, MIP nanoparticles formed a relatively stable and homogeneous suspension of nano-sized particles of ~150 nm diameter (Figure 2). The integration of the MIP in paper membranes was carried out by simple impregnation with the suspension of MIP nanoparticles. Subsequent immersion of the membrane in an acetic acid bath led to concomitant template removal and precipitation of the MIP nanoparticle suspension in the form of MIP NPAs that attached to the cellulose fibers (Figure 4). A screening of membrane materials showed that chromatographic paper membranes were the most adequate for the incorporation of the MIP NPA compared with other materials such as polyethylenesulfone or glass fiber membranes.

**Figure 5:**
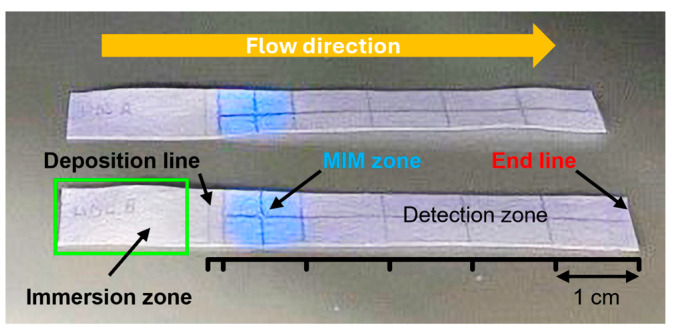
Photograph of two MIM-containing integrated strips used for HDL extraction under UV light illumination. The strip was sub-divided into three zones. The central MIM zone contained MIP NPAs attached to cellulose fiber. The presence of the MIP was made visible by its blue fluorescence upon UV illumination. The sample deposition zone was used for the addition of the lipoprotein-containing samples on a line in front of the MIM (deposition line) and was partially immersed in a buffer bath (immersion zone) for the elution through the strip. Finally, a detection zone served for the quantification of the unextracted lipoproteins. The elution by the buffer was stopped when it reached the end line.

To assess the extraction performance of the MIM for HDL, a specific section of a 7 cm × 1 cm chromatographic paper strip was functionalized with MIP NPAs. This modified strip was divided into three zones (Figure 5):

1. A sample deposition zone—Where the lipoprotein-containing sample was applied at the 17 mm position;
2. An MIM zone—Located between 20 and 30 mm. The MIM zone contained ~0.28 mg of MIP NPAs deposited by dispensing drops of MIP NPA solution at the 25 mm mark. Assuming complete retention of the MIP NPAs during preparation (which remains unverified), this would correspond to an estimated density of ~47 mg/mL—approximately eight times higher than in the depletion experiment shown in Figure 3;
3. A detection zone—Positioned downstream for monitoring unextracted lipoproteins.

The MIM zone was easily visualized under UV illumination due to the inherent blue fluorescence of the MIP NPAs. The integrated strip functioned similarly to thin-layer chromatography. Initially, the sample was placed in the deposition zone just before the MIM zone. Subsequently, the front edge of the strip was dipped into a buffer reservoir to start capillary-driven elution. The buffer front carried the lipoproteins through the MIM zone towards the detection area (Figure 5), with complete migration usually happening within 7–8 min. It is worth noting that the capillary flow noticeably slowed while moving through the MIM zone. During elution, the MIP NPAs stayed confined within the MIM, as evidenced by the lack of blue fluorescence outside this area.

### 3.4. Performance of the MIM for Solid-Phase Affinity Extraction of HDL

**Figure 6:**
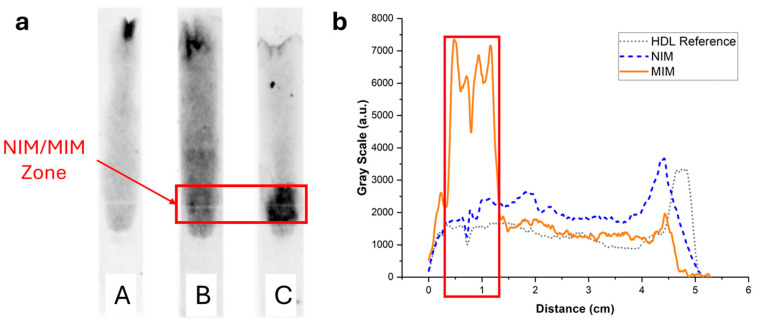
(**a**) Typical fluorescence images of integrated paper strips after elution of a solution containing 7.5 µg of HDL-C labeled with Nile Red. Darker tunes are associated with higher concentrations of Nile Red and, consequently, concentrations of lipoproteins. (**b**) Background-corrected fluorescence intensity profiles along the strip corresponding to strips A (grey dotted line), B (blue dashed line), and C (orange solid line). Strip A was a reference strip containing no MIM. Most HDL eluted all the way to the end line with some background fluorescence, either due to non-specific adsorption of HDL to the membrane or non-specific coloring by Nile Red. Strip B contained an MIM functionalized with NIP NPAs. The intensity profile was almost identical to strip A. Finally, strip C contained an MIM functionalized with MIP NPAs. A clear retention of HDL was observed both in the image and the intensity profile, demonstrating SPAE of HDL.

To image and quantify HDL in paper strips, the lipoproteins were stained with the lipophilic fluorescent dye Nile Red, and the strips after elution were imaged with a luminescence imaging system. In the absence of MIM or in the presence of an MIM containing non-binding non-imprinted polymer nanoparticle aggregates (NIP NPA), HDL (7.5 µg corresponding to the amount typically present in a drop of 25 µL of blood) eluted mostly unretained up to the end line (Figure 6 (strips A and B)). Some residual fluorescence was observed to spread over the paper membrane, which could be due to unspecific binding of HDL to the membrane and/or Nile Red non-specific coloration. In the presence of MIP NPA in the MIM zone, a strong retention of HDL was clearly observed (Figure 6, strip C). Integration of the fluorescence signal was very much dependent on the interpretation of the origin of the observed background fluorescence (Figure 6b). If all the background fluorescence was due to non-specific staining by Nile Red, integration indicated that ~92% of HDL was retained in the MIM zone. On the other hand, if all the background fluorescence was due to non-specific binding of HDL to the paper membrane during migration, the integration yielded ~52% retention of HDL in the MIM zone. In any case, retention of HDL in the MIM zone (~52% to ~92%, average of ~72%) was very efficient.

**Figure 7:**
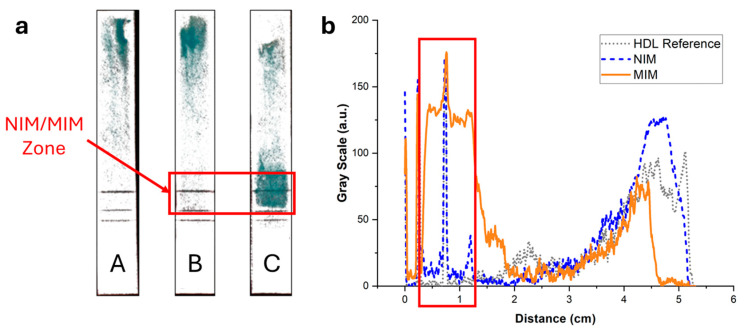
(**a**) Images of the strips of Figure 6 after spraying with a reactive enzymatic solution. The intensity of the green color is proportional to the cholesterol content. (**b**) Background-corrected fluorescence intensity profiles corresponding to strips A (grey dotted line), B (blue dashed line), and C (orange solid line). Strip A contained no MIM; most of the HDL eluted all the way to the end of the strip, with some cholesterol spread along the strip. Strip B contained an MIM functionalized with NIP NPAs. The intensity profile was almost identical to the profile of strip A, demonstrating that no unspecific bindingof HDL to NPA occurred. Finally, strip C contained an MIM functionalized with MIP NPAs. A clear retention of HDL-C in the MIM zone was observed, with some cholesterol present outside of the zone.

To exclude potential artifacts caused by the staining with Nile Red, the elution of HDL in integrated strips was imaged using an enzymatic colorimetric test as an orthogonal technique. A green color proportional to the cholesterol content developed upon addition of the enzymatic reagents. Figure 7 displays the same integrated strips as in Figure 6 after treatment with the reactive solution. The same trend was observed, with no retention of HDL when the strips did not contain an MIM (Figure 7, strip A) or contained an MIM functionalized with NIP NPAs (Figure 7, strip B). Furthermore, a strong retention in the MIM zone was observed when the MIM was functionalized with MIP NPAs (Figure 7, strip C). The background was much less intense than for the images obtained with Nile Red staining, which indicates that Nile Red might have led to non-specific staining. Integration of the color signal (Figure 7b) showed that typically 64% of HDL-C was retained within the MIM zone in the presence of MIP NPAs. The effective retention is likely higher because ~10% of the cholesterol content in the HDL sample was free cholesterol (data from the manufacturer). Given the difficulty in integrating image profiles, the values obtained using the enzymatic colorimetric test and Nile Red staining are not significantly different, and it can be estimated that the actual retention efficiency lies between the two obtained values, i.e., between 64 and 72% (average of 68%).

Overall, this set of data demonstrates the efficiency of SPAE of HDL in an integrated strip, which could be further developed into a complete point-of-care assay.

### 3.5. Elution Profile of LDL in the MIM-Containing Strips and Performance of the LDL-C Assay

**Figure 8:**
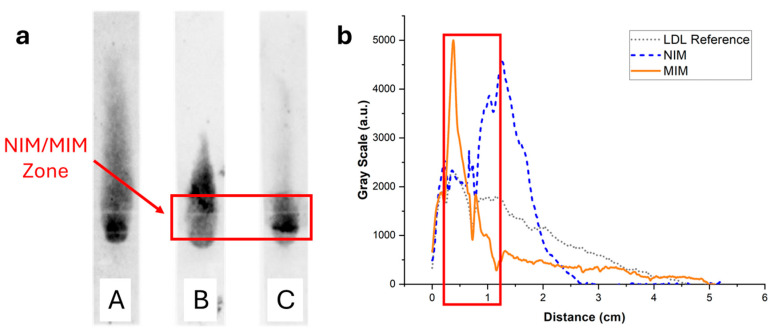
(**a**) Typical fluorescence images of integrated paper strips after elution of a solution containing 7.5 µg of LDL-C labeled with Nile Red. Darker tunes are associated with higher concentrations of Nile Red and, consequently, concentrations of lipoproteins. (**b**) Background-corrected fluorescence intensity profiles along the strip corresponding to strips A (grey dotted line), B (blue dashed line), and C (orange solid line). Strip A was a reference strip containing no MIM. LDL was interacting non-specifically with the membrane and was spread all along the strip. Strip B contained an MIM functionalized with NIP NPAs. The retention close to the MIM was stronger compared with strip A, demonstrating non-specific retention of LDL in the MIM. Finally, strip C contained an MIM functionalized with MIP NPAs. Non-specific retention of LDL in the MIM was observed, similarly to strip B, but to a higher extent.

**Figure 9:**
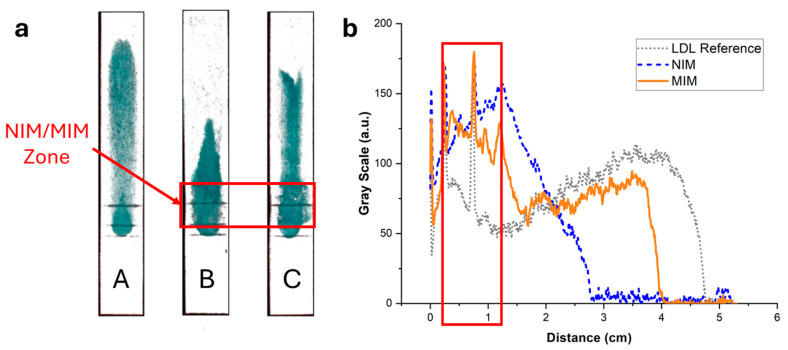
(**a**) Images of the strips of Figure 8 after spraying with a reactive enzymatic solution. The intensity of the green color is proportional to the cholesterol content. (**b**) Background-corrected fluorescence intensity profiles corresponding to strips A (grey dotted line), B (blue dashed line), and C (orange solid line). Strip A contained no MIM; LDL non-specifically adsorbed to the paper membrane during the flow, resulting in a broad distribution of cholesterol. Strip B contained an MIM functionalized with a non-imprinted polymer NPA. A stronger retention was observed close to the MIM compared with strip A, demonstrating non-specific retention of LDL in the MIM. Finally, strip C contained an MIM functionalized with MIP NPAs. Non-specific retention of LDL in the MIM was observed, similarly to strip B, but to a lower extent.

The elution of LDL through the MIM-containing strips was investigated using the same methodology as used for HDL, using staining with Nile Red or enzymatic coloring. In the simple strips containing no MIM, LDL (7.5 µg, like HDL) stained with Nile Red clearly bound non-specifically to the paper membrane, resulting in LDL being spread all along the strip (Figure 8, strip A). This was confirmed by imaging with the enzymatic assay (Figure 9, strip A), where it was apparent that a portion of LDL did not even elute and was retained at the deposition spot. In the presence of NIP NPAs (Figure 8, strip B) or MIP NPAs (Figure 8, strip C) in the MIM zone, LDL accumulated in the deposition spot and extended over into the first portion of the detection zone. Together with the images recorded using the enzymatic assay (Figure 9, strips B and C), no significant difference was apparent between the MIM prepared using the NIP and the MIP NPAs. The accumulation of LDL in the MIM zone in the presence of an MIM might have been due to either increased interaction with the membrane as the flow rate was slowed down within the MIM zone, to non-specific adsorption of LDL to the NIP and MIP NPAs, or a combination of both effects.

The non-specific adsorption had a negative impact on the quantification of LDL-C in the detection zone using the enzymatic assay (Figure 5 and Figure 9). Only ~60% of LDL-C was detected in the detection zone, with ~40% remaining entrapped in the MIM zone. This was, however, still enough to assure adequate sensitivity for the quantification of LDL-C in the physiological range. Despite the non-specific binding, the MIM enriched LDL compared with HDL prior to testing with a selectivity factor, defined as the mass ratio of HDL-C to LDL-C remaining in the MIM, of ~1.6. However, the profile of elution of LDL in the strip varied from experiment to experiment, resulting in variability of the selectivity factor and of the amount of recovered LDL-C in the detection zone. This made the test unsuitable for accurate point-of-care testing due to insufficient reproducibility and robustness.

**Figure 10:**
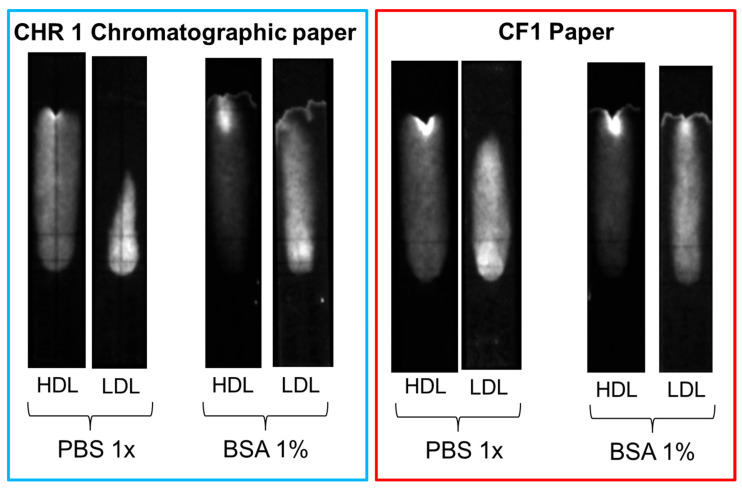
Comparison of non-specific binding of HDL and LDL on different paper substrates (CHR01 chromatographic paper and dipstick CF1 paper) with and without the addition of 1% BSA in the eluent.

Several strategies were explored to increase the mobility of LDL and avoid non-specific binding, such as the addition of surfactants (e.g., Tween 20) or BSA to the eluent and the replacement of the chromatographic paper with other membrane materials. Slightly better results were obtained when the chromatographic paper, Whatman CHR01, was replaced with Cytiva CF1 dipstick paper and 1% BSA was added to the eluent solution. However, none of the adopted strategies proved successful in completely displacing LDL beyond the MIM zone in the reference (without deposition of MIP nanoparticles) samples (Figure 10). The non-specific binding of LDL seemed, therefore, to be the result of the inherent instability of LDL, as the propensity of LDL to bind to surfaces, in particular negatively charged surfaces, is well known [30,31]. Non-specific interactions of LDL with cellulose substrates have been reported in specific contexts, such as with macroporous cellulose beads [32] and carboxymethyl cellulose beads [33]. However, to the best of our knowledge, the non-specific adsorption of LDL to cellulose fibers in lateral flow membranes remains a little-explored property.

## 4. Conclusions

A novel way to produce MIM by precipitation of MIP nanoparticles inside a paper membrane was used for solid-phase affinity extraction of HDL-C in a paper-based point-of-care assay. The efficiency of the MIM was estimated to be between 64% and 72% (on average 68%), depending on the measurement method. It is interesting to note that the efficiency of the MIM appeared better than the efficiency of the MIP NPAs in solution as ~68% of 7.5 µg of HDL-C was retained by the ~0.28 mg of MIP NPAs deposited in the membrane (uptake capacity of ~18 µg of HDL-C/mg of MIP) within the few minutes that it took for the flow to pass through the membrane. These results are remarkable given the complex nature of the HDL lipoprotein analyte. The high density in MIP NPAs in the MIM, a better distribution of the MIP NPAs thanks to their adhesion to the membrane fibers, and a closer contact between MIP NPAs and HDL within the pores of the paper membrane might be potential explanations for the improvement in uptake capacity. These reasons may also partly explain the strong non-specific binding of LDL, which was primarily caused by the paper membrane itself, as shown by the results in the absence of MIM. The MIM further aggravated this inherent non-specific binding. Nevertheless, we believe that this novel method for straightforwardly preparing MIMs might be of particular interest for other point-of-care diagnostic applications with less complex analytes, such as small molecules or proteins.

## Data Availability

The data presented in this article can be obtained from the authors upon request.

## Supplementary Materials

The following supporting information can be downloaded at: https://www.mdpi.com/article/10.3390/bios15100685/s1, Table S1: Visual effect of the additions of various monomers and chemicals to an HDL solution.

## Abbreviations

The following abbreviations are used in this manuscript:

ABTS	2,2′-Azino-bis(3-ethylbenzothiazoline-6-sulfonic acid) diammonium salt
APS	Ammonium persulfate
BSA	Bovine serum albumin fraction V
CE	Cholesterol esterase
CO	Cholesterol oxidase
DHEBA	*N*,*N*-(1,2-dihydroxyethylene)bisacrylamide
HDLs	High-density lipoproteins
HDL-C	High-density lipoprotein-cholesterol
HRP	Horseradish peroxidase
LDLs	Low-density lipoproteins
LDL-C	Low-density lipoprotein cholesterol
MAA	Methacrylic acid
MIM	Molecularly imprinted membrane
MIP	Molecularly imprinted polymer
NPAs	Nanoparticle aggregates
NIP	Non-imprinted polymer
PBS	Phosphate buffer saline
POC	Point of care
SPAE	Solid-phase affinity extraction
TEMED	*N*,*N*,*N*’,*N*’-tetramethylethylenediamine
VP	1-vinyl-2-pyrrolidone
