# Supplementary material for: A Molecularly Imprinted Membrane for High-Density Lipoprotein Extraction in Point-of-Care Testing
Source: Biosensors (Basel). 2025 Oct 10;15(10):685. doi: 10.3390/bios15100685 (PMC12563577; doi:10.3390/bios15100685)
Supplement: Supplementary file 1 [file biosensors-15-00685-s001.zip › biosensors-3845556-supplementary.pdf]

biosensors

Supplementary Material

Electronic Supporting Information: A Molecularly Imprinted Membrane for High-Density Lipoprotein Extraction in Point-of-Care Testing

Gian Luca de Gregorio [1], Denis Prim [1], Alberto Zavattoni [2], Italo Mottini [2], Daniele Pezzoli [2], Federico Roveda [2], Marc E. Pfeifer [1] and Jean-Manuel Segura [1,*]

[1] Institute of Life Sciences - School of Engineering, HES-SO // University of Applied Sciences and Arts Western Switzerland, 1950 Sion, Switzerland
[2] PRIMA Lab SA, Balerna, Switzerland
* Correspondence: jmanuel.segura@hevs.ch (J.-M.S.)

A) Screening of the affinity of monomers for HDL

A screening of monomers for their affinity for HDL was performed by supplementing them to a solution of HDL and observing the apparition of turbidity indicative of interactions between the monomer and HDL (see Table S1). This screening confirmed that MAA and VP displayed a stronger affinity for HDL compared with most other common monomers. Only 4-vinylpyridine (4-VP) exhibited a similar interaction.

Table S1: Visual effect of the additions of various monomers and chemicals to an HDL solution. VP = 1-vinyl-2-pyrrolidinone, HMAAm = N-hydroxymethyl acrylamide, NIPAm = N-isopropyl acrylamide, TBAm = tert-butyl acrylamide, MAA = methacrylic acid, 4VPy = 4-vinylpyridine, AAm = acrylamide, DHEBA = N,N′-(1,2-dihydroxyethylene)-bisacrylamide, MBA = N,N'-Methylenebisacrylamide, EtOH = ethanol, and PEGMA = poly(ethylenglycol)methacrylate.

Reagent	Effect	Reagent	Effect
VP	Cloudiness	Sodium methacrylate	No effect
HMAAm	No effect	Acrylic acid	Cloudiness
NIPAm	No effect	DHEBA	No effect
TBAm	No effect	MBA	No effect
MAA	Coagulation	HCl	Increased viscosity
Acetic acid	Increased viscosity	EtOH	Increased viscosity
4VPy	Coagulation	PEGMA	No effect
AAm	No effect		
